# Vocal complaint in physical education teachers and its association with the cardiovascular system

**DOI:** 10.1186/1755-7682-7-7

**Published:** 2014-02-26

**Authors:** Denise S Cunha, Luiz Carlos de Abreu, Ana C F Frizzo, Marco A Cardoso, Vitor E Valenti

**Affiliations:** 1 Departamento de Fonoaudiologia, Faculdade de Filosofia e Ciências UNESP, Av. Hygino Muzzi Filho, 737, 17525-900 Marilia SP, Brasil; 2 Departamento de Morfologia e Fisiologia, Faculdade de Medicina do ABC, Av. Príncipe de Gales, 821, 09060-650 Santo Andre, SP, Brazil; 3 Programa de Pós-Graduação em Fisioterapia, Faculdade de Ciências e Tecnologia, UNESP, Rua Roberto Simonsen, 305, 19060-900 Presidente Prudente, SP, Brasil

**Keywords:** Functional performance, Health, Neuroscience, Teaching

## Abstract

**Background:**

We examined the vocal complaints and evaluated the correlation between the vocal handicap index (VHI) and heart rate variability (HRV) in physical education teachers. We evaluated 46 teachers.

**Method:**

The subjects were investigated regarding voice complaint and the VHI was applied. HRV was recorded at seated rest for ten minutes and it was analyzed in the time, frequency domains, geometric indices and fractal exponents. The three domains of the VHI were correlated with the indices of HRV.

**Results:**

The physical education teachers presented a VHI score much below the standard of the physiological normality. There was correlation of the organic domain of the VHI with the NN50 and pNN50 and correlation of the functional domain and organic domain of the VHI with the HF index of HRV.

**Conclusion:**

The physical education teachers evaluated reported vocal complaints that affected their function and it is suggested to be related with the cardiac autonomic regulation.

## Background

The voice command of the physical education teacher influences the student discipline, raising the level of awareness and understanding the performance if the activities proposed. The use of voice is important to encourage and motivate them to continue the execution of the exercise [1,2]. It is essential to have good health vocal to generate a psychodynamic vocal that contributes to the affirmation of such aspects.

It is known that the work environment of the physical education teacher presents multiple risk factors to vocal health, with high risk of vocal health impairments and dysphonia, once they are submitted to a specific vocal demands associated with physical movements [3-6].

The use of voice depends on the respiratory system [7] and the breath is related with the cardiac autonomic regulation. A non-invasive method for investigation of autonomic nervous system (ANS) that describes the oscillations of the intervals between consecutive heartbeats is the heart rate variability (HRV). This method is a conventionally accepted term to describe the fluctuations in the intervals between consecutive heartbeats (RR intervals), which are indicated to influence the sinusal node. The methods used for HRV analysis include the geometric, time and frequency domain methods [8].

Vocal complaint among physical education teachers is a variable related to impairment in quality of life and a stressful factor [9]. In addition, HRV is also a method that analysis stressful conditions [10]. Thus, we evaluated the vocal complaints and investigated the association between the vocal handicap index and HRV in physical education teachers.

## Method

### Study population

We analyzed 46 physical education teachers, all volunteers were informed about the procedures and objectives of the study and, after agreeing, have signed a term of informed consent. All procedures were approved by the Ethics Committee in Research of the Faculty of Sciences of the Universidade Estadual Paulista, Campus of Marilia (Protocol No. CEP-2012-382) and followed the resolution 196/96 National Health 10/10/1996.

### Exclusion criteria

We considered the following exclusion criteria: cardiopulmonary disorders, neurological and other impairments that prevent the subject to perform the procedures, and treatment with drugs that influence cardiac autonomic regulation.

### Vocal handicap index (VHI)

It is an instrument that assesses vocal problems, it presents 30 questions, 10 for each domain: functional, organic and emotional domains. The calculation of the score is done by sum of all questions for each domain. The overall score ranges from 0 to 12 points in the sum and 0–40 in each domain, with the higher the score, the more intense the perceived voice handicap. The final results are converted to a scale base 100, using a simple rule of three (Behlau et al.,) [11].

### Experimental protocol

Data were collected in our laboratory under controlled temperature (21°C–25°C) and humidity (50%–60%), and volunteers were instructed to avoid consuming alcohol, caffeine and substances that influence the ANS for 24 hours before evaluation. Data were collected between 8 a.m. and 12 a.m. in order to minimize the interference of circadian rhythm. All procedures necessary for the data collection were explained to the individuals, and the subjects were instructed to remain at rest and to not talking during the data collection.

After the initial evaluation the heart monitor strap was placed on each subject’s thorax over the distal third of the sternum. The HR receiver (Polar RS800CX monitor, Polar Electro OY, Kempele, Finland) was placed on the wrist.

The subject remained 10 minutes seated at rest with spontaneous breathing.

### HRV analysis

The R-R intervals recorded by the portable HR monitor (with a sampling rate of 1000 Hz) were downloaded to the Polar Precision Performance program (v. 3.0, Polar Electro, Finland). The software enabled the visualization of HR and the extraction of a cardiac period (R-R interval) file in "txt" format. Following digital filtering complemented with manual filtering for the elimination of premature ectopic beats and artifacts, at least 256 R–R intervals were used for the data analysis. Only series with more than 95% sinus rhythm was included in the study. We evaluated the linear and non-linear indices of HRV. For calculation of the indices we used the HRV Analysis software (Kubios HRV v.1.1 for Windows, Biomedical Signal Analysis Group, Department of Applied Physics, University of Kuopio, Finland) [12].

### Linear indices of HRV

To analyze HRV in the frequency domain, the low frequency (LF = 0.04 to 0.15 Hz) and high frequency (HF = 0.15 to 0.40 Hz) spectral components were used in ms^2^ and normalized units (nu), which represents a value relative to each spectral component in relation to the total power minus the very low frequency (VLF) components, and the ratio between these components (LF/HF). The spectral analysis was calculated using the Fast Fourier Transform algorithm [13].

The analysis in the time domain was performed by means of SDNN (standard deviation of normal-to-normal R-R intervals), the total number of adjacent RR intervals with a difference of duration greater than 50 ms (pNN50), the percentage of adjacent RR intervals with a difference of duration greater than 50 ms (pNN50) and RMSSD (root-mean square of differences between adjacent normal RR intervals in a time interval) [13].

### Geometric indices of heart rate variability

HRV analysis was performed by means of geometrical methods: RRtri, TINN and Poincaré plot (SD1, SD2 and SD1/SD2 ratio). The RRtri was calculated from the construction of a density histogram of RR intervals, which contains the horizontal axis of all possible values of RR intervals measured on a discrete scale with 7,8125 ms boxes (1/128 seconds) and on the vertical axis, the frequency with which each occurred. The union of points of the histogram columns forms a shape like a triangle. The RRtri was obtained by dividing the total number of RR intervals used to construct the histogram by their modal frequency (RR interval value that most frequently appeared on RR) [8].

The TINN consists of the measure of the base of a triangle. The method of least squares is used to determine the triangle. The RRtri and the TINN express the overall variability of RR intervals [8].

The Poincaré plot is a map of points in Cartesian coordinates, constructed from the values of RR intervals obtained, where each point is represented on axis x (horizontal/abscissa) by the previous normal RR interval, and on axis y (vertical/coordinate), by the following RR interval.

For quantitative analysis of the plot, an ellipse was fitted to the points of the chart, with the center determined by the average RR intervals, and the SD1 indexes were calculated to measure the standard deviation of the distances of the points to the diagonal y = x, and SD2 measures the standard deviation of the distances of points to the line y = - x + RRm, where RRm is the average of RR intervals. The SD1 is an index of instantaneous recording of the variability of beat-to-beat and represents parasympathetic activity, while the index SD2 represents HRV in long-term records, and reflects the overall variability. Their ratio (SD1/SD2) shows the ratio between short and long variations of RR intervals [8,14].

The qualitative analysis of the plot was made through the analysis of the figures formed by its attractor, which were described by Tulppo et al. [15] in:

Figure in which an increase in the dispersion of RR intervals is observed with increased intervals, characteristic of a normal plot.

Small figure with beat-to-beat global dispersion without increased dispersion of RR intervals in the long term. We used the software HRV analysis.

### Fractal analysis of HRV

For the analysis of the fractal properties of the heart rate, detrended fluctuation analysis (DFA) was applied to a time series of the R–R intervals obtained from the participants. The procedure for the calculation of DFA is made up of the following steps:

The R–R series obtained experimentally is integrated using the expression:

$$Y\left(k\right)=\overset{k}{\underset{i=1}{\sum }}\left[\text{RR}\left(i\right)-\text{RRave}\right]$$

in which Y(k) is the k-th term of the integrated series (k = 1, 2,…, N); R–R(i) is the i-th value of the R–R intervals; and R–R_ave_ is the mean of the R–R intervals of the original series, with N length:

$$\text{RRave}=\frac{1}{N}\overset{N}{\underset{i=1}{\sum }}\text{RR}\left(i\right)$$

The integrated time series is then divided into intervals with a length of n, (n = 1, 2,…, N). In each of these intervals, the local trend of the series is calculated by a straight line of minimum squares adjusted to the data. The y-coordinate of this straight line was denominated Yn(k). The integrated series was then detrended [Y(k)], subtracting the local tendency Yn(k) in each interval. For a given interval of size n, the size characteristic of the fluctuation for the integrated and detrended series is calculated by:

$$F\left(n\right)=\sqrt{\frac{1}{N}\overset{N}{\underset{K=1}{\sum }}{\left[Y\left(k\right)-\text{Yn}\left(k\right)\right]}^{2}}$$

This procedure is repeated for all intervals of size *n*, thereby obtaining a relation between the mean of the fluctuations [*F*(*n*)] and the size of the intervals (*n*). A linear relation on a log–log graph indicates a scale exponent law, based on the following formula:

$$F\left(n\right)\approx \text{na}$$

in which *α* is the scale exponent, which can be calculated by linear regression on a log–log graph (16). The following were calculated: short-term fractal exponent (alpha-1), corresponding to a period of 4 to 11 beats; long-term fractal exponent (alpha-2), corresponding to periods longer than 11 beats; and the alpha-1/alpha-2 ratio [16].

When α = 0.05, there is no correlation and the signal consists of white noise; if α = 1.5, the signal resembles random walk (Brownian motion); and if 0.5 < α < 1.5, there are positive correlations.

### Statistical analysis

Standard statistical methods were used for the calculation of means and standard deviations. Normal Gaussian distribution of the data was verified by the Shapiro-Wilk goodness-of-fit test (z value >1.0). For parametric distributions we applied the correlation Pearson test. For non-parametric distributions we used the Spearman test. Differences were considered significant when the probability of a Type I error was less than 5% (p < 0.05). We used the Software GraphPad StatMate version 2.00 for Windows, GraphPad Software, San Diego California USA.

## Results

The study population was composed of 46 physical education teachers, regarding gender, 46% (21) were male and 54% (25) were female and the average age of the group was 43 years (24–64 years old). We observed that most teachers (38%) had workday between 31 and 40 hours per week and in average they were working in two schools.

We noted 67% (14) males and % (18) women presented vocal complaints during the last four years. When asked regarding the cause of vocal disorders, 62% (13) male and 60% (15) female reported that it was due to intensive use of the voice, whereas 33% (7) male and 48% (12) female reported due to stress.

By contrast, we observed that although most teachers reported vocal complaints, only 10% (2) were male and 24% (6) female perform some kind of treatment. The type of treatment was contested over the use of medication, in both genders, with males at 10% (2) and 16% in females (4).

The most reported symptoms in the male group were: 57% (12) hoarseness and 62% (13) dry throat. In the female group, the most related symptoms were: 60% (15) hoarseness, 56% (14) failed in voice, 52% (13) effort to speak, 92% (23) dry throat, 68% ( 17) hoarseness, 56% (14) sore throat and 52% (13) secretion/phlegm in the throat.

According to Table 1 we observe the total scores of the physical education teachers based on each domain.

**Table 1:** Mean scores of the participants regarding the vocal handicap index protocol

**VHI**	**Scores**
Emotional	3.28
Functional	4.63
Organic	8.91
Total	16.82

Regarding the VHI domains and the time domain indices of heart rate variability, there was a weak but significant correlation between the domain 2 (organic) and the NN50 and pNN50 indices. However, there was no correlation of the VHI domains with the RMSSD, SDNN indices and mean HR and RR interval (Table 2).

**Table 2:** Correlation between the VHI domains and the time domain indices of heart rate variability

	**Functional**		**Organic**		**Emotional**	
	**r**	**p**	**r**	**p**	**r**	**p**
**Mean RR**	-0.01	0.96	-0.25	0.28	-0.30	0.20
**Mean HR**	0.02	0.93	0.25	0.28	0.33	0.16
**SDNN**	0.17	0.47	-0.21	0.37	0.09	0.70
**RMSSD**	0.30	0.21	-0.30	0.20	-0.17	0.48
**NN50**	0.13	0.57	-0.53	0.01	-0.04	0.86
**pNN50**	0.10	0.67	-0.53	0.01	-0.05	0.82

VHI: Vocal handcap index.

In relation to the VHI domains and the frequency domain indices of heart rate variability we observed in Table 3 a weak but significant correlation between the VHI domain 1 (Functional) and the HF index in absolute units (ms^2^) and correlation between the VHI domain 2 (Organic ) and the HF index in absolute units (ms^2^). On the other hand, there was no correlation between the VHI domains with the LF index in normalized and absolute units, HF index in normalized and absolute units and the LF/HF ratio.

**Table 3:** Correlation between the VHI domains and the frequency domain indices of heart rate variability

	**Functional**		**Organic**		**Emotional**	
	**r**	**p**	**r**	**p**	**r**	**p**
**LF (ms**^**2**^**)**	0.05	0.83	-0.20	0.39	0.048	0.84
**LF (nu)**	-0.20	0.39	0.047	0.84	-0.03	0.87
**HF (ms**^**2**^**)**	0.30	0.04	-0.34	0.04	0.02	0.92
**HF (nu)**	0.23	0.32	-0.03	0.89	0.01	0.94
**LF/HF**	-0.21	0.38	0.04	0.87	-0.03	0.89

VHI: Vocal handcap index.

Based on Table 4, there was no correlation between the VHI domains and the geometric indices of heart rate variability (TINN, RRtri, SD1/SD2, SD1 and SD2).

**Table 4:** Correlation between the VHI domains and the geometric indices of heart rate variability

	**Functional**		**Organic**		**Emotional**	
	**r**	**p**	**r**	**p**	**r**	**p**
**SD1**	0.12	0.61	-0.26	0.26	0.05	0.81
**SD2**	0.15	0.52	-0.15	0.52	0.13	0.57
**SD1/SD2**	0.05	0.82	-0.20	0.40	-0.14	0.56
**RRTri**	0.14	0.54	-0.27	0.25	0.08	0.73
**TINN**	0.14	0.55	-0.15	0.53	0.24	0.31

VHI: Vocal handcap index.

According to Table 5, there was no correlation between the VHI domains and the fractal exponents of heart rate variability (Alpha-1/Alpha-2, Alpha-2 and Alpha-1).

**Table 5:** Correlation between the domains of VHI and the exponents of fractal heart rate variability

	**Functional**		**Organic**		**Emotional**	
	**r**	**p**	**r**	**p**	**r**	**p**
**Alpha-1**	-0.05	0.80	0.11	0.62	0.05	0.81
**Alpha-2**	0.12	0.59	0.20	0.39	0.14	0.54
**Alpha-1/Alpha-2**	-0.07	0.75	0.00	0.99	-0.06	0.79

VHI: Vocal handcap index.

## Discussion

Considering the use of voice by physical education teachers during their work routine, it was hypothesized that this population would present voice injuries associated with stress regarding the autonomic function. In this study we endeavored to evaluate the association between the vocal handicap index and HRV in physical education professors. According to the VHI, the physical education teachers evaluated presented decreased voice perception. As a main finding we reported negative association between the parasympathetic indices of HRV and some components of the vocal handicap index.

The results of our study suggest that women present more interference factors of working conditions and job stress on their vocal health. This finding confirms what has already been described in the literature, where women are more prone to vocal disorders [17], because it is an area of work in which they are greater number and also due to the routine at school with students and administrative tasks at home. The age found in this study showed us that the renovation has taken place over the last few years on public schools in Brazil. However, despite the short time working and being young, some teachers have already vocal complaints, although it tends to be observed in older teachers. This justifies the complaint to last more than four years in large part of the population.

The calculation of the VHI score is done by sum of all questions for the three domains. The higher the score, the more intense the perceived voice handicap and the final results are converted to a scale base 100, using a simple rule of three [11]. To our surprise, the mean of the physical education teachers total score was below 20, which indicates the bad condition of the voice that was reported by the volunteers investigated. This result may be explained by the fact that the teacher is required to make use of voice command in order to influence the discipline of the students, the level of comprehension and understanding to perform the activities proposed. As the room is not always disciplined, the exacerbated use of voice sometimes causes stress [1]. These factors have been reported as the cause of the voice disorder and are directly linked to the daily habits of these professionals evaluated in this study.

In this context, habits such as screaming, speaking too much in open place, talking during overcharged activities and talking during physical activities performance were listed as risk factors for vocal disorders [17]. Most teachers in our study reported that drink water during class, however, such an act was not observed by the researcher during the study. When asked about guidance on precautions to prevent vocal disorders, the physical education teachers reported that never received. This fact shows us the importance of education as the best use of voice over work tasks. The profession in which the professional have his/her voices for his/her work, should have in their curriculum, at least one course that addressed aspects of vocal hygiene, vocal health, health promotion and prevention of general vocal. It would positively interfere in the execution of physical education teacher quality of life.

Despite vocal complaints, the physical education teachers reported satisfaction with their voice. This may suggest that teachers do not have parameters for a vocal self-perception or are not aware of their vocal problem. This is evident when we noted in our study that there were few teachers who seek treatment when his/her voice is altered and when they seek this treatment is sometimes medication. This might lead us to think of self medication, since the demand for specialized professional when they were with the altered voice was very low.

Considering that HRV is a method used to evaluate cardiovascular [18] and respiratory disorders [14,16], stress responses that are related to the cardiac autonomic regulation [10] we also investigated the association of the fractal exponents, time, frequency and geometric indices of HRV with the VHI score of the physical education teachers. We found no association between the VHI and the geometric indices and fractal exponents of the HRV. On the other hand, there was negative association of the functional and organic domains of the VHI with the time-domain indices and the frequency-domain index of HRV that correspond to the parasympathetic activity on the heart [19-21].

Based on our data, there was negative association between the NN50 and pNN50 indices of HRV and the domain 2 of the vocal handicap index. The time domain indices of HRV NN50 and pNN50 represent the parasympathetic activity on the heart while the domain 2 of the vocal handicap index represents the organic domain, with the higher the score, the more intense the perceived voice handicap. This data suggests that the higher the parasympathetic activity on the heart the less intense the perceived voice handicap of the physical education teachers. The perceived voice in this case is related to the anatomic structure of the voice components, such as the ones surrounding the larynx. In this context, a previous study reported intensification in speaking rate which was indicated by a decrease in the length of phonation in males and females subjects during the cold pressor test [22]. Considering that the cold pressor test is an autonomic test that increases the sympathetic nervous system activity [23], the authors [22] suggested that increased sympathetic activity induces phonation length. These data do not support the negative association between parasympathetic indices of HRV and the domain 2 of the vocal handicap index. We believe that this is due to the vocal complaints reported by the physical education teachers, which may influence the autonomic nervous system.

The relationship between the larynx anatomical structure and the parasympathetic activity on the heart found in our study is also supported by the fact that bronchodilation induced by decreased parasympathetic activity and sympathetic arousal is suggested to result in increased transglottal airflow, increasing the speed at which the vocal folds are closed and also increasing the voice power [24]. Furthermore, it was previously reported that heart rate is involved in around 10-12% of the total vocal intensity perturbation measure. The authors suggested that this modulation could result in part from the influence of the heart rate on the subglottal pressure [25]. The mentioned studies explain the involvement of the parasympathetic nervous system activity in voice mechanisms, which may also support the association between vocal complaint and the cardiac autonomic regulation.

Our study presents a point that is worth to be raised. We did not evaluate a control group, because our focus was not to investigate the cardiac autonomic regulation in physical education teachers, we suggest a further study to evaluate this issue with more details.

## Conclusion

Voice disorders in the physical education teachers investigated shows no major impact on their lives due to a vocal self-perception that is not appropriate. The vocal complaint reported by the physical education teacher is possibly associated with the cardiac autonomic regulation. We strongly suggest an implementation of prevention programs related to voice disorder in schools directed to the physical education teachers.

## Competing interest

The authors declare that they have no competing interest.

## Authors’ contributions

DCS, LCA and VEV draft the manuscript and determined the study design. DCS, ACFF and VEV performed statistical analysis. All authors reviewed the final version of the manuscript and approved it.
